# Terpenoid Variations within and among Half-Sibling Avocado Trees, *Persea americana* Mill. (Lauraceae)

**DOI:** 10.1371/journal.pone.0073601

**Published:** 2013-09-09

**Authors:** Jerome Niogret, Nancy D. Epsky, Raymond J. Schnell, Edward J. Boza, Paul E. Kendra, Robert R. Heath

**Affiliations:** USDA-ARS, Subtropical Horticulture Research Station, Miami, Florida, United States of America; Instituto Valenciano De Investigaciones Agrarias, Spain

## Abstract

Chemical analyses were conducted to determine the qualitative and quantitative differences in monoterpenes and sesquiterpenes in plant material from avocado trees, *Persea americana* Mill. (Lauraceae). The initial study analyzed plant material sampled from the trunk to the leaves through different branch diameters to quantify proximo-distal spatial differences within a tree. All trees were seedlings initiated from a single maternal tree. Two-way analysis of variance was conducted on 34 chemicals that comprised at least 3% of the total chemical content of at least one tree and/or location within a tree. There were significant interactions between genotype and location sampled for most chemicals. Parentage analysis using microsatellite molecular markers (SSR's) determined that the four trees had three fathers and that they represented two full-siblings and two half-sibling trees. Descriptive discriminant analysis found that both genotype and location within a tree could be separated based on chemical content, and that the chemical content from full-siblings tended to be more similar than chemical content from half-siblings. To further explore the relationship between genetic background and chemical content, samples were analyzed from leaf material from 20 trees that included two sets of full-sibling seedling trees, the maternal tree and the surviving paternal tree. Descriptive discriminant analysis found good separation between the two full-sibling groups, and that the separation was associated with chemistry of the parental trees. Six groups of chemicals were identified that explained the variation among the trees. We discuss the results in relation to the discrimination process used by wood-boring insects for site-selection on host trees, for tree selection among potential host trees, and the potential use of terpenoid chemical content in chemotaxonomy of avocado trees.

## Introduction

Plants display a large diversity of secondary metabolites, among which terpenoids are the largest class, with approximately 50,000 structurally identified [1]. Terpenoids, primarily C_10_ monoterpenes and C_15_ sesquiterpenes, are known to play an important role in the biology and ecology of plants, directly or indirectly influencing their interactions with the environment. Plants generally produce complex mixtures of terpenoids that may differ greatly among species. For individuals of the same species, these mixtures frequently differ in the proportion and amount of each chemical compound, providing distinct chemical phenotypes [2]. Qualitative and quantitative differences can also be seen between different developmental stages and the chemical profiles may vary in different tissues and organs of a plant [3]. Therefore, differences in the chemical profiles are also expected in different tissues and at different times within an individual. As a result, plant populations exhibit a large amount of phenotypic chemical variation in terpenoid content. The levels and spatial distribution may influence the capacity of herbivores and pathogens to adapt and exert selection based on the presence or concentrations of plant chemicals [4]–[6]. Terpenoids are known to play various roles in the plant kingdom. They have been identified to be responsible for attraction of insects to host plants [7], [8]. Others are toxic or may repel herbivore attacks [9]–[11]. Terpenoids can also be emitted from herbivore-damaged plants to attract natural enemies and indirectly confer plant defense [12].

The cambial tissue, defined as the lateral meristem including the vascular cambium and cork cambium in a vascular plant, carries the secondary metabolites (often several major compounds accompanied by derivatives and minor components), from organ to organ within a tree [13]. There are changes in the proportion of specific terpenoids at different locations within a tree, resulting from the accumulation, transformation and/or emission of these terpenoids. For example, some bark beetles (Coleoptera: Curculionidae: Scolytinae) find their host trees by attraction to host volatiles from a distance [14], but may locate the most appropriate site for entry/feeding/reproduction within a tree based on spatial differences in chemical profiles. Consequently, the patterns of phenotypic variation in plant secondary metabolites have a strong influence on plant-herbivore interactions and are important factors in understanding the interactions in natural populations. Variation of plant chemical phenotypes within a population can be explained by a combination of genetic [15], developmental [16] and environmental [17] factors and the interaction of all three. The age structure, the environmental heterogeneity, and the limits in gene flow in a natural population are important factors that will determine the variability and the spatial structure of the secondary chemistry landscape of plants. In order to obtain an accurate description of the distribution of secondary metabolites and the natural variability among trees, controlling those factors becomes essential.

Avocado, *Persea americana* Mill. (Lauraceae), has been subdivided into three horticultural races: Mexican [*P. americana* cv. *drymifolia* (Schect. & Cham.) Blake], Guatemalan (*P. americana* cv. *guatemalensis* Wms.) and West Indian (*P. americana* cv. *americana* Mill.). The West Indian race is known to be from the lowland areas of the Pacific coast of Central America, while the Guatemalan and Mexican races are native to specific highland areas of their respective countries [18]. In this study, we evaluated seedling avocado trees of the same age and grown under the same environmental conditions. Using a combination of chemical analysis of the terpenoid content of seedling trees grown from seeds of a known maternal parent, and genetic analysis with microsatellite molecular markers to determine paternal parent, we addressed the following questions: Are qualitative and quantitative spatial differences present in the chemical phenotype in avocado trees? How do the terpenoids differ spatially from the trunk through the branches to the leaves? Does the variability in chemical profiles allow for an accurate separation between half- and full-sibling avocado trees?

## Materials and Methods

The seedling avocado trees used in this study were part of a late fruiting population that were evaluated for fruit quality and productivity as part of an avocado selection program at the USDA-ARS Subtropical Horticulture Research Station (Miami, FL). The female parent was the cultivar ‘Melendez’ (added to SHRS germplasm collection in 1966). ‘Melendez’ is a late maturing variety selected in Puerto Rico. It has a medium oil content, rich-yellow flesh and tight seed, and is a West Indian × Guatemalan hybrid [19], [20]. Seeds from this tree were harvested and germinated in January 1995, and placed into the field in June 1995. All seedlings were planted in a rectangular plot (20×30 m), and thus were grown under the same cultural and environmental conditions (i.e., Krome soil: Loamy-skeletal, carbonatic, hyperthermic lithic udorthents). The ‘Melendez’ tree was open pollinated, so the paternal parent of each seedling was not known.

For the initial study on spatial differences in chemical content, we sampled randomly 4 seedling trees that remained from the selection study. Trees were sampled over a three month period (June to August 2009) when there was an average temperature of 29.1±1.2°C and relative humidity of 74.2±6.7%. For the subsequent study on the relationship between genetic background and chemical content, trees were selected based on their genetic background and availability. Trees sampled included the maternal tree (Melendez), a paternal tree (Waldin), eight Melendez × Waldin seedlings (full-siblings) and ten Melendez × ‘General Bureau’ seedlings (full-siblings). By the time of this study, the pollen parent ‘General Bureau’ had died and no other representative of that cultivar was available for sampling. For both studies, samples were collected between 10 am and 12 pm on sunny days to reduce potential effects of environmental conditions on chemical content.

### Parentage analysis: Microsatellite markers and PCR amplification

Avocado trees are open-pollinated, thus most reproduction is by outcrossing. Distance from the maternal parent is therefore an important factor as pollination is mainly made by insects. DNA was isolated from leaf material following a previously published method [21]. In summary, DNA extraction was performed on leaf tissue using the Fast DNA kit (BIO 101, Inc; Carlsbad, CA) and a cell disrupter (FastPrep FP 120; Savant Instruments, Inc.; Holbrook, N.Y.). DNA was then quantified on a spectrophotometer (DynaQuant 200; Amersham Pharmacia; Piscataway, Calif.). Microsatellite markers were selected based on amplification consistency and level of polymorphism, and PCR amplication reactions were carried out following the same protocol as described previously [21]. Initially, samples included the original maternal parent ‘Melendez’, 40 potential male parents that were growing within 50 meter proximity of the maternal ‘Melendez’ tree (*i.e*., the most likely pollen donors), and the four seedlings used in the spatial analysis study. Subsequently, all seedlings were sampled to determine full-and half-sibling family groups. The microsatellite markers were developed previously [22] and used in a genetic diversity study [20]. Of those 14 microsatellite markers, 13 (AVAG11 was excluded) were used for parentage analysis of the ‘Melendez’ seedlings. PCR amplification reactions and capillary electrophoresis was similar to previously published work [21]. Parentage analysis was performed using the program CERVUS Ver. 3.0.3 [23], [24]. This software uses a simulation program to generate log-likelihood scores and provides a confidence statistic for assigning paternity. Simulations were accomplished using 100,000 simulated offspring, 40 potential fathers and a 0.75 proportion of candidate fathers sampled. ‘Melendez’ was used as the known parent and the other 40 cultivars, including ‘Melendez,’ were candidate pollen parents.

### Plant Materials and Sample Preparation

Plant material was collected using methods reported previously [25]. Previous studies showed that no qualitative differences in chemical content were detected among intact avocado branches, newly-damaged branches, and newly rasped bark samples from those branches, leading to the assumption that production of chemicals was not induced at detectable levels during the time period evaluated (30 min) [26]. Crook et al. [27] demonstrated an increased production of several sesquiterpenes after mechanically injuring green ash trees, *Fraxinus pennsylvanica*. However, elevated levels of those induced compounds were not observed until 24 h after tree injury.

Trunk and branch samples included bark and underlying cambium layers that were obtained by manual rasping with a microplaner. Trunk samples were obtained at approximately 60 cm height where tree circumference was 53.5±2.1 cm. Intact branches were cut from trees to provide leaf, petiole and branch samples. Leaves were separated from petioles and cut into sections (1.2 cm^2^), petioles were rasped. Branch samples were obtained from sequential sections (each 9 cm long) of a branch, labeled A (proximal to trunk) to H (distal from trunk) according to the branch diameter: A: 3.80±0.2, B: 2.60±0.80, C: 2.30±0.80, D: 1.90±0.10, E: 1.30±0.03, F: 0.80±0.10, G: 0.60±0.03, H: 0.50±0.04 cm, respectively. There were three sample replicates obtained per location per tree for chemical analysis in the spatial study, with each seedling treated as a genotype replicate. There were two sample replicates of leaves obtained per tree in the genetic background study, with the maternal, paternal, and each sibling family group treated as separate genotype groups.

### Chemical Collections and Analysis

Plant substrates (6 g per sample) were placed into beakers sealed with Parafilm M (Thomas Scientific, Swedesboro, NJ) and held at room temperature for 1 hr prior to sampling. Volatile chemicals were sampled by Solid Phase Microextraction (SPME) with a 100 µm poly-dimethylsiloxane coating (non-bonded) fiber (Supelco, Bellefonte, PA). Fibers were inserted through a small hole in the parafilm and exposed to headspace volatiles for 2 min. Samples were manually shaken during each SPME chemical collection. For the spatial study, adsorbed chemical compounds were analyzed using a gas-chromatograph (ThermoQuest Trace GC-FID 2000, Austin TX). The column consisted of fused silica, 25 m long, 0.25 mm I.D, with DB-5MS phase, film thickness 0.25 μm (J&W Scientific, Agilent Technologies, Santa Clara, CA). The GC temperature was programmed from 50–130°C at 15.0°C min^−1^, then from 130–220°C at 10.0°C min^−1^, and then held at 220°C for 4 min. Chemicals were identified by their Kovats Retention Index (RI). For the genetic study, samples were analyzed using a shorter fused silica DB-5 column (J&W Scientific, Agilent Technologies, Santa Clara, CA), 10 m long, 0.18 mm I.D, with film thickness 0.18 μm. The GC temperature was programmed from 50°C for 1 min, 50 to 220°C at 35°C min^−1^, 220°C for 2 min. Use of this temperature program and the column type resulted in faster analysis but with similar results and accuracy.

All chemical identifications were conducted with a GC-mass spectrometer (Agilent Technologies 5975B, Santa Clara, CA, USA) with a DB-5MS column. The GC-MS temperature was programmed from 40–80°C at 16.0°C min^−1^, then from 80–230°C at 7.0°C min^−1^, and then held at 230°C for 10 min. Volatile chemicals were identified based on the comparison of mass spectra with the NIST Mass spectral program version 2.0d and NIST/EPA/NIH mass spectral library (NIST11), or by comparison with the following standards: α-cubebene (Bedoukian Research Inc., Danbury, CT, USA), α-copaene (Fluka Analytical, Stenheim, Germany), α-humulene and β-caryophyllene (Sigma Chemical Co., St Louis, MO, USA), (-)-alloaromadendrene (Sigma Chemical Co., St Louis, MO, USA), δ-cadinene (Sigma Chemical Co., St Louis, MO, USA).

### Statistical analysis

For each sample, the relative amount of each compound was calculated as a percentage of the whole blend. Only chemicals representing >3% of the total chemical content in at least one location (spatial study) or in one tree (genetic background study) were included in the data analysis. For evaluation of chemical blend from different locations of the tree, comparisons were made for five of the tree sections, specifically leaf, petiole, distal branch section (H), proximal branch sample (A) and trunk. Two-way analysis of variance (ANOVA) with interaction were conducted using Proc GLM (SAS Institute, 2008) to determine effects of location within a tree and tree genotype (replicate) on chemical content, with separate analysis for each chemical. Due to significant interactions for most of the chemicals, the factors of tree genotype and tree location were combined to produce one classification variable, genotype/tree location (20 levels), with percentage of each chemical used as quantitative variables in descriptive discriminant analysis [28]. Stepwise discriminant analysis using Proc STEPDISC was used to determine which chemicals explain most of the variation among groups [29]. These chemicals were then retained and canonical discriminant analysis using Proc CANDISC (SAS Institute, 2008) was used as a dimension reduction technique to find the linear combinations of the chemicals (canonical correlations) that gave the best separation among the classification variables. Scatterplots of class means produced by canonical correlation 1 (*x* axis, the classification that produces the best discrimination among the groups) versus canonical correlation 2 (*y* axis, the classification that gives the second best discrimination) were used to visually summarize the separation based on chemical content of groups as specified by the classification variable.

Two-way ANOVA using a mixed model with tree genotype as a qualitative factor and average branch diameter as a quantitative factor (Proc GLM) was used to determine if there were relationships between branch diameter (sections 1–8) and percentage of chemicals that were common for all genotypes. Thus, chemicals for which there was no interaction between genotype and branch diameter were used for regression analysis, tested for fit to linear and logarithmic regression models (Statview 5.0.1, SAS Institute, Cary, NC).

## Results

### Genetic analysis for parental verification

No mother-offspring mismatching loci were found between ‘Melendez’ and the seedlings, verifying the maternal parent for all seedlings. Three different pollen parents were identified for the four seedlings sampled in the spatial study, all with no mismatching loci. Progeny 1 and progeny 2 are full-siblings both having ‘General Bureau’ as the father (LOD = 3.73E+00 and 9.58E+00, respectively). Progeny 3 and progeny 4 had ‘Wilson Popenoe’ and ‘Waldin’ identified as the putative fathers (LOD = 5.46E+00 and 4.55E+00, respectively). One trio locus mismatch was found for the parents of progeny 1; however, the trio delta score provided 95% confidence in the correct parentage assignment.

A total of 7 pollen parents were identified as putative fathers for the seedling trees. ‘General Bureau’ and ‘Waldin’ were the most common fathers, with 10 and 8 seedlings confirmed with high confidence, respectively (Table 1). The full-siblings were treated as replicates, with mean percentage chemical per tree used for statistical analysis. Of the remaining trees, there were 1–6 progeny per father with 0–1 progeny per father confirmed with high confidence. Therefore, no other family groups were samples. Chemical content of the maternal ‘Melendez’ and the paternal ‘Waldin’ were determined and sample per tree was used as replicate for statistical analysis.

**Table 1:** Summary results of the paternal parentage analysis using microsatellite markers on Melendez offspring.

Tree (Progeny)	Putative father: Waldin (LOD score)	Tree (Progeny)	Putative father: General Bureau (LOD score)
1	5.75E+00*	1	6.55E+00*
2	5.83E+00*	2	6.42E+00*
3	5.67E+00*	3	7.16E+00*
4	6.21E+00*	4	6.55E+00*
5	6.09E+00*	5	5.64E+00*
6	5.33E+00*	6	7.30E+00*
7	6.34E+00*	7	7.90E+00*
8	6.05E+00*	8	7.28E+00*
		9	7.28E+00*
		10	7.28E+00*

The log-likelihood ratio (LOD) compares the likelihood of an individual being the parent of a given offspring divided by the likelihood of these two individuals being unrelated. * Delta scores for 95% confidence for assigned parentage.

### Spatial chemical variation within trees

**Figure 1:**
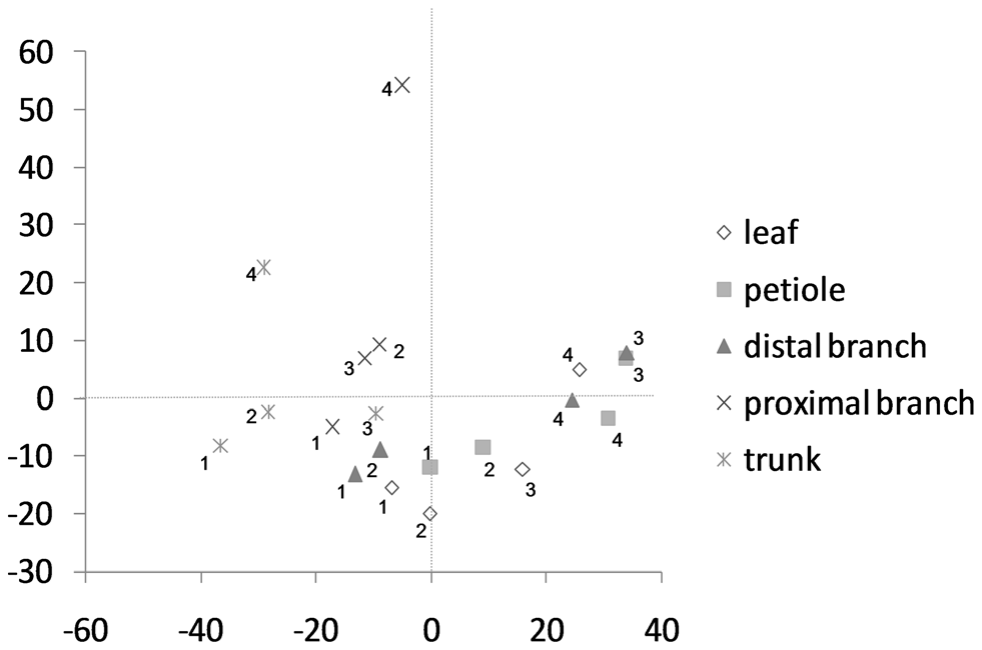
Canonical discriminant analysis on the chemicals from full-sibling (1, 2) and half-sibling (3, 4) avocado trees. Locations sampled included leaf, petiole, distal branch section (0.5 cm diam), proximal branch section (3.8 cm diam) and trunk (n = 3 samples per tree per location).

There were 34 chemical compounds detected by GC in which each comprised at least 3% of the total terpenoid content in at least one genotype or one location (Table 2). Stepwise discriminant analysis selected 26 of these chemicals for subsequent canonical discriminant analysis of the classification groups. A scatterplot of the class means produced by plotting canonical correlation 1 versus canonical correlation 2 (i.e., the linear combinations of the 26 chemicals that gave the best and second best discrimination among the classification groups) allowed visualization of the chemical profile for each group (Fig. 1). The class means of chemicals from trunks and sections of the branch closest to the trunk (proximal end) were all negative for canonical correlation 1. The class means of chemicals from the leaves, petioles and section of branch closest to the petiole (distal end) were predominantly positive for canonical correlation 1, although there were a few means (2 leaves, 1 petiole and 2 branch distal ends) that were ≤0 for canonical correlation 1. For four of the five locations, chemical content from the full-siblings (progeny 1 and progeny 2) tended to be closer together than the profiles from their half-siblings (progeny 3 and progeny 4). The exception was chemicals from the proximal end of the branches, for which progeny 2 and progeny 3, half-siblings, were the closest.

**Table 2:** Chemicals that comprised >3% of volatile chemicals from plant material collected from four avocado trees (‘Melendez’ seedlings, n = 3 per location per tree).

		RI	used in CDA	Relative amount (%)				
	Molecules			Leaf	Petiole	Distal branch	Proximal branch	Trunk
								
1	Monoterpene	816	y	8.4±15.2	0.8±1.3	0.0±0.0	0.1±0.2	0.0±0.0
2	Monoterpene	868	y	10.1±8.1	0.3±0.3	0.2±0.1	0.0±0.0	0.0±0.0
3	Monoterpene	877	y	1.2±2.8	3.2±4.1	2.1±3.8	0.3±0.2	0.0±0.0
4	Sabinene^1^	954	y	10.6±6.1	1.2±0.7	1.0±1.3	3.5±3.5	2.81±1.94
5	β-pinene*	988	y	4.4±3.5	0.4±0.5	0.4±0.8	0.0±0.0	0.4±0.2
6	Monoterpene	999	y	7.6±5.6	1.2±0.7	1.0±1.2	2.1±1.6	1.9±1.5
7	Monoterpene	1010	y	0.8±1.7	0.1±0.2	0.0±0.0	0.0±0.0	0.0±0.0
8	Monoterpene	1022	n	0.0±0.1	0.6±1.4	0.2±0.3	0.0±0.0	0.0±0.0
9	3-carene*	1025	y	0.0±0.0	1.1±1.4	0.5±1.0	7.4±7.3	6.7±5.0
10	Monoterpene	1052	y	3.8±1.6	1.7±0.6	0.6±0.6	1.3±0.6	1.6±1.0
11	Monoterpene	1124	n	0.5±1.5	0.1±0.1	0.0±0.1	0.1±0.1	0.4±0.1
12	Monoterpene	1208	y	0.5±1.7	0.0±0.0	0.0±0.0	0.0±0.0	0.0±0.0
13	Monoterpene	1290	n	0.5±1.5	0.1±0.3	0.3±0.6	0.1±0.1	0.1±0.1
14	δ-elemene	1347	y	1.3±0.8	2.6±1.0	2.0±1.6	6.5±4.1	6.3±3.2
15	α-cubebene*	1360	y	4.0±2.9	2.1±1.3	9.7±6.1	13.2±5.5	16.9±7.9
16	Sesquiterpene	1386	y	0.1±0.1	0.2±0.2	1.0±1.2	2.9±2.8	3.3±4.0
17	α-copaene*	1392	y	1.9±1.1	1.9±0.3	5.4±2.1	13.8±6.6	15.4±7.2
18	β-elemene	1402	y	4.4±2.1	6.0±1.4	8.1±2.8	7.9±1.6	10.1±4.8
19	Sesquiterpene	1409	y	0.0±0.0	0.0±0.0	0.4±0.8	0.2±0.1	0.3±0.4
20	(*Z*)-α-bergamotene	1423	y	0.2±0.4	0.3±0.3	1.0±1.0	0.5±0.6	1.5±1.1
21	Sesquiterpene	1436	y	0.0±0.0	1.9±1.8	29±1.8	1.3±2.1	0.7±0.9
22	β-caryophyllene*	1441	y	26.8±10.8	33.6±11.0	17.4±4.4	11.0±7.1	6.8±3.8
23	(*E*)-α-bergamotene	1448	y	1.2±1.5	3.4±0.6	3.6±1.8	0.9±0.6	0.5±0.3
24	Sesquiterpene	1456	y	0.4±1.0	0.0±0.0	0.1±0.2	0.7±0.6	0.5±0.5
25	Sesquiterpene	1470	y	0.2±0.3	1.5±0.5	1.8±1.3	0.2±0.2	0.1±0.1
26	α-humulene*	1476	y	1.9±0.7	2.9±1.1	2.4±1.1	1.4±0.9	0.9±0.4
27	Alloaromadrendrene*	1480	y	0.1±0.1	1.5±1.3	1.3±1.2	4.9±3.2	4.5±3.0
28	Muurolene	1489	y	0.1±0.1	0.3±0.1	0.8±0.6	2.3±1.5	2.4±2.4
29	β-cubebene	1499	y	4.3±2.7	23.2±5.4	25.5±11.4	4.4±3.2	2.1±2.2
30	Sesquiterpene	1504	n	0.0±0.0	0.2±0.2	0.9±2.6	0.0±0.0	0.0±0.0
31	Sesquiterpene	1508	y	0.5±0.8	1.1±1.6	0.6±0.9	0.0±0.0	0.1±0.2
32	Sesquiterpene	1510	y	0.0±0.0	0.0±0.0	0.2±0.3	1.4±0.9	1.3±1.3
33	β-bisabolene	1519	y	0.2±0.3	1.3±0.4	1.4±1.1	0.1±0.1	0.1±0.1
34	δ-cadinene*	1529	y	0.5±0.3	0.9±0.2	1.6±0.7	3.6±2.0	3.5±1.6

Chemical inclusion in canonical discriminant analysis (CDA) is designated by yes (y) or no (n). RI: Retention index calculated on DB5-MS. *: Identification based on comparison with standards.

1 : Identification based on comparison with NIST 11 mass spectra library and published reports (Sagrero-Nieves 1995).

**Figure 2:**
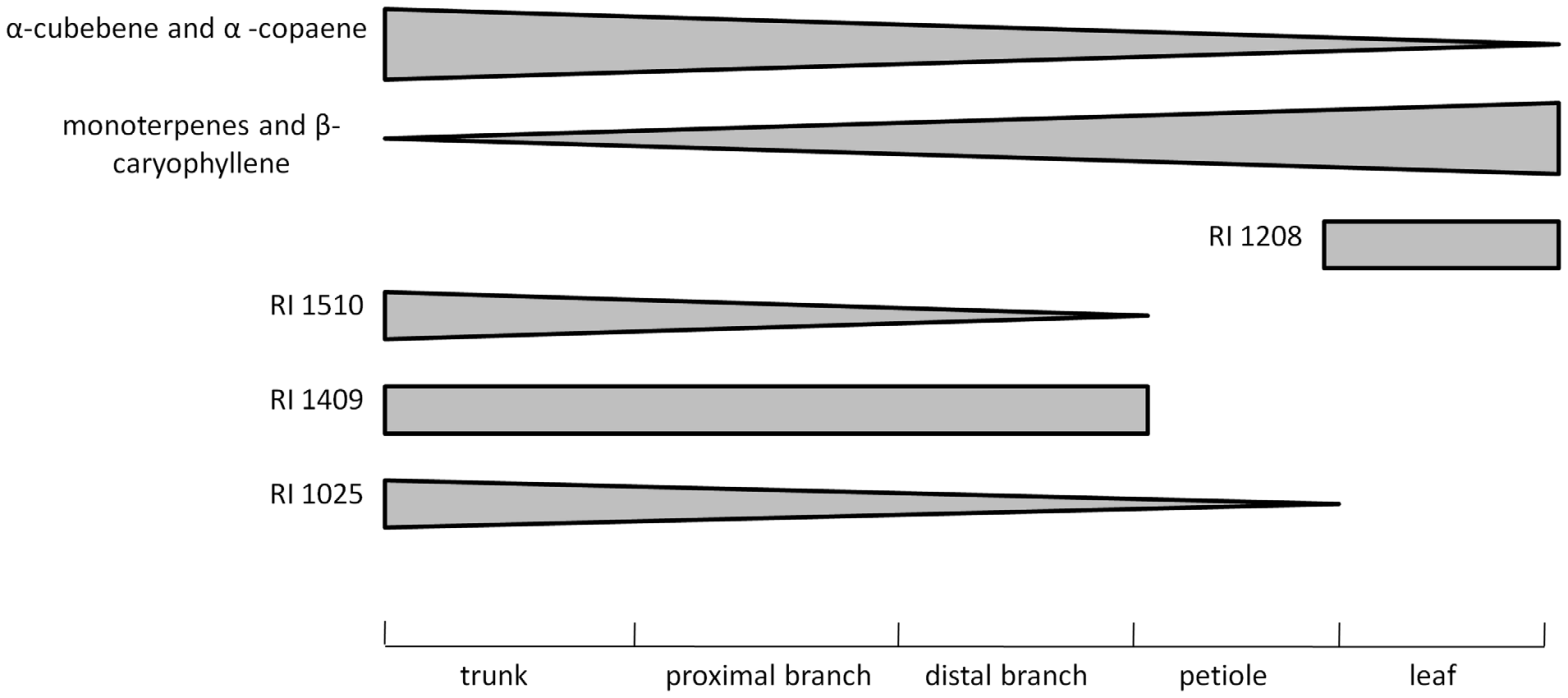
Schematic illustration of the distribution of selected chemicals according to their relative proportions. RI = Retention Index. Trunks were sampled at 60 cm height; proximal and distal branches correspond to 3.8 and 0.5 cm diameter respectively.

Fifteen chemicals (56%) were present in all samples (trunk, branch sections, petioles and leaves). Only chemicals RI = 1510 and 1409 were specific to the trunk and branches (absent from the petiole and leaves), compound RI = 1025 was present in all samples except in the leaves. Chemical compound RI = 1208 was the only compound specific to the leaves. No chemical compound was found to be specific of the petioles or the trunk alone. Six monoterpenes (RI = 816, 868, 877, 954, 988, and 999) were predominant in the leaves compared to other locations. Chemical RI = 1441 (identified as β-caryophyllene) was predominant in both petioles and leaves, and also high in branches and trunks. Chemicals RI = 1360, 1392 (identified as α-cubebene, α-copaene, respectively) and 1402 were predominant in the wood material, e.g. both distal and proximal branch samples, as well as in the trunk (Fig. 2).

Regression analysis was used to evaluate the relationship between chemical percentage and diameter of a branch, which was divided into eight 9 cm long sections that ranged from 0.5–3.8 cm average diameter. Thirteen chemicals, which had the same pattern among all four genotypes, were used for this analysis. There was no change in percentage with increase in diameter for two of the chemicals (RI = 1360 (α-cubebene) and 1347; *t* = 1.48 and −0.02; *P* = 0.143 and 0.83, respectively), α-cubebene was the major chemical in the trunk samples, remaining high in all of the branch sections. For five of the chemicals, there was a direct relationship between increase in diameter and increase in percentage of the chemical (RI = 954, 999, 1052, 1347, 1392; *t* = 3.40, 2.41, 3.45, 4.87 and 4.66; *P* = 0.001, 0.02, 0.001, 0.0001 and 0.0001, respectively). Of those chemicals, only α-copaene (RI = 1392) (possibly co-eluted with ylangene) was present in high percentages in the trunk. The remaining chemicals decreased with the increase in branch diameter, and the decrease was best fit by a logarithmic curve (1441 (identified as β-caryophyllene), 1448, 1470, 1476 (identified as α-humulene), 1499, and 1519; *t* = −4.494, −8.16, −6.454, −3.9, −9.018 and −7.60; *P* = 0.0001, 0.0001, 0.0001, 0.0002, 0.0001, and 0.0001, respectively). Among these chemicals, compound RI = 1499 was the most abundant in the small branch sections and petioles, while β-caryophyllene (RI = 1441) was the most abundant in the petioles and leaves.

### Chemical differences and genetic background

**Figure 3:**
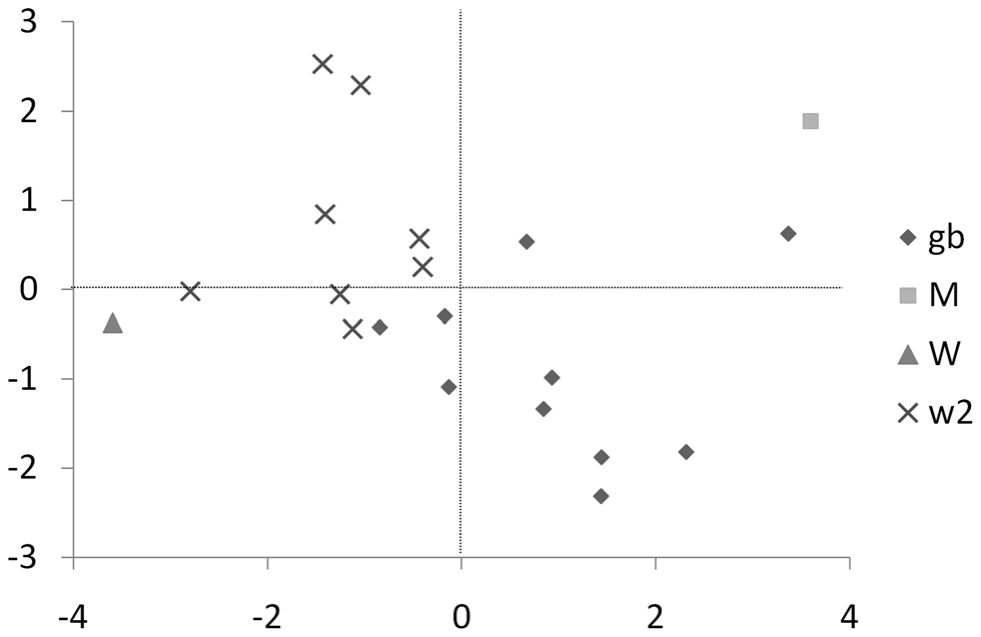
Canonical discriminant analysis of the leaf chemicals from two sibling populations with different paternal parentages. W2  =  Waldin siblings (N = 8 trees); gb  =  General Bureau siblings (N = 10 trees); M  =  Melendez (n = 2 samples per tree, maternal parent of all siblings); W  =  Waldin (n = 2 samples per tree, paternal parent of Waldin siblings).

Sampling leaves is the least damaging and easiest sampling method in term of cost of time and effort, compared to rasped wood samples. For these reasons, we decided to investigate the correlation between chemical phenotypes and genetic backgrounds among two half-sibling populations (N = 8 and 10 siblings for ‘Waldin’ and ‘General Bureau’ offspring, respectively) using leaf samples. Leaf samples were also obtained from the maternal parent ‘Melendez’ and the surviving paternal parent ‘Waldin.’ Chemical analysis found fifteen compounds that comprised more than 3% of the leaf chemical content. Canonical discriminant analysis selected five groups of chemical compounds that explained the separation among the genotypic groups (Fig. 3). The groups of chemical compounds were classified (from 1 to 5) by their importance to explain the separation among the genotypic groups. The chemical groups were group 1: RI = 1365, 799, 858, 870, 943, 981, 986, 1040, 1207, 1395, 1408, 1444, 1481, 1506, and 1521; group 2: RI = 1207, 858, 870, 943, 986, 1365, 1408, 1444, 1481, and 1521; group 3: RI = 1481, 799, 858, 870, 943, 986, and 1207; group 4: RI = 1506, 799, 981, 1040, 1365, 1395, and 1408; group 5: RI = 1395, 799, 858, 870, 1365, 1408, 1444, 1481, 1506. Amounts (%) of representative chemicals are given in Table 3, and these chemicals are correlated with the remaining chemicals in each group. The representative chemicals included α-cubebene, β-caryophyllene, α-humulene, an unknown chemical, and α-copaene. Together, these chemicals represented about 33, 22, 35, and 52% of the whole chemical profile for the ‘Melendez’ × ‘Waldin’ siblings, the ‘Melendez’ × ‘General Bureau’ siblings, ‘Melendez’ and ‘Waldin’, respectively. The two full-sibling populations clustered according to their genetic background (Fig. 3). It is interesting to note that every ‘Melendez’ × ‘Waldin’ seedling was distributed between their mother ‘Melendez’ and their father ‘Waldin.’. Unfortunately, similar information could not be obtained for this relationship for the ‘General Bureau’ family.

**Table 3:** Average composition (%) of the representative compounds of each group of chemicals selected by the stepwise discriminant analysis.

Group	Representative chemical (RI^1^)	Name^2^	General Bureau siblings	Waldin siblings	Mother Melendez	Father Waldin	F^3^	P^3^
								
1	1365	α-cubebene	2.0±1.5	2.3±1.8	1.4±0.2	5.7±1.2	9.21	0.001
2	1444	β-caryophyllene	13.1±5.2	24.2±14.4	25.3±5.6	37.3±4.4	4.14	0.027
3	1481	α-humulene	1.4±0.4	2.5±1.2	2.4±0.4	3.5±0.7	3.78	0.035
4	1505	unknown	3.6±0.9	3.1±1.0	4.2±0.6	3.4±0.5	3.71	0.038
5	1395	α-copaene	1.4±0.6	1.4±0.5	2.1±0.0	1.8±0.5	2.93	0.070

Groups are numbered from 1 to 5 depending on their degree of explanation of the variation among sibling populations.

1 Retention Indexes calculated on DB-5 column.

2 Verified with synthetic compounds.

3 STEPDISC procedure Stepwise selection among chemicals between General Bureau (N = 10) and Waldin (N = 8) siblings.

## Discussion

Little is known about the mechanisms underlying rejection of non-host tree species by wood-boring beetles. Rejection could be based on a lack of host volatile characteristics or the presence of repellent or deterrent stimuli [10], [11], [30], [31]. In contrast, many herbivorous insects have evolved very specific behavioral responses to volatile chemicals that signal the presence of a host [7], [8], [14], [32]. Secondary metabolites emitted by host plants are often used as kairomonal cues for long-range location of host trees; however, host volatiles may also be used for short-range location of appropriate sites to initiate feeding, boring, and/or oviposition. Finding a specific site on a host plant may be directed by the variations in terpenoid chemistry that occur along the proximo-distal axis of the host tree. For example, there are several pest beetle species that attack specific parts of host avocado trees. The Fuller’s rose weevil, *Pantomorus cervinus* (Boheman) (Coleoptera: Curculionidae), chews on leaf margins during the adult stage [33]. Species such as *Melalgus confertus* LeConte (Coleptera: Cerambycidae) [33] and *Xylosandrus compactus* (Eichhoff) (Coleoptera: Curculionidae) [34], [35], are branch and twig borers. The latter species, the black twig borer, is restricted to branches 1–3 cm in diameter [34]. The entrance holes of the redbay ambrosia beetle, *Xyleborus glabratus* Eichhoff (Coleoptera: Curculionidae) have been observed to be more numerous on the trunk and large diameter branches of host trees [36].

In this study, several terpenoid compounds were observed to occur along proximo-distal gradients within the avocado tree. The sesquiterpene β-caryophyllene increased in proportion from the trunk to the small diameter branches, to reach its highest proportion in the petioles and leaves. It has been demonstrated that β-caryophyllene is attractive to several foliage-feeding species, including the boll weevil, *Anthonomus grandis* Boh. (Coleoptera: Curculionidae) [37]. In addition, some wood-boring beetles respond positively to β-caryophyllene, including *Scolytus intricatus* (Ratz.) (Coleoptera: Curculionidae) and the emerald ash borer, *Agrilus planipennis* Fairmaire (Coleoptera: Buprestidae) [27]. Both *S. intricatus* and *A. planipennis* also respond positively to α-copaene [27], another sesquiterpene widely distributed within the plant kingdom. This compound was found in high levels in the trunk of avocado, and decreased in concentration with the reduction of branch diameter to reach its smallest proportions in the petioles and leaves. α-Copaene is suspected to be the primary host-based attractant for the redbay ambrosia beetle [38], and as mentioned previously, females of *X. glabratus* target the trunk and large branches of host trees, sites where α-copaene levels are the highest. In field tests with freshly-cut avocado bolts and essential oil lures, captures of *X. glabratus* were positively correlated with substrate emissions of this sesquiterpene [26], [39], [40]. Several studies have investigated the ultrastructure, composition and distribution of oil cells in the Lauraceae family, including avocado trees, using light, fluorescence and electron microscopy. In this plant family, oil cells are the primary site for essential oil biosynthesis, secretion and storage [41]. Essential oils are concentrated liquids containing volatile secondary metabolites. The oil cells are commonly present in roots, stems, bark, fruits and leaves in Lauraceae trees [41]–[45], and are also observed in all tissues analyzed in *Persea americana* [46]. This distribution of oil cells, with probable tissue-specific variability in abundance and composition, could explain the correlations found in our study among several mono- and sesquiterpenes all along the proximal-distal axis. By percent composition, the major chemical observed in the trunk of avocado was α-cubebene (∼17% of total chemicals), and its levels remained fairly constant (∼11%) within branch sections from 3.8 to 0.6 cm diam. This compound is a known attractant for two species of wood-boring beetle, *Scolytus pygmaeus* (F.) and *S. laevis* (Chapuis) (Coleoptera: Curculionidae) [27], [47]. Due to the high levels of α-cubebene in the trunk and large branches of avocado, this sesquiterpene should also be evaluated as a potential kairomone for *X. glabratus*.

The avocado variety ‘Melendez’ is a hybrid between West Indian and Guatemalan races, while ‘General Bureau,’ ‘Wilson Popenoe’ and ‘Waldin’ are pure West Indian race cultivars. Commercial avocado cultivars are, in general, highly heterozygous based on molecular marker analysis, and segregation for chemical traits should be expected among seedlings of commercial clones. However, highly homozygous individuals have been identified among clones within each cultivar background [20], [21]. In several cases it is apparent that ancestral members of a group evolved the biosynthetic capacity to produce certain secondary metabolites. The analysis of secondary metabolite profiles in Fabaceae, Solanaceae, Lamiacaeae and other plant families, showed that a shared chemical characteristic present in almost all members of a monophyletic could be used as taxonomic markers [3]. The absence of such a trait in phylogenetically derived groups is probably due to differential gene expression (the genes are present in the genome but not necessarily expressed) [3]. In our study, the genetic relatedness of progeny 1 and 2, which are full-siblings, may explain both the qualitative similarities observed in the chemical phenotypes between both trees, and the differences with progeny 3 and 4. Progeny 4, the ‘Melendez’ × ‘Waldin’ hybrid, had both qualitative and quantitative chemical patterns in the proximal end of the branch and the trunk that were quite different from the two full-siblings (progeny 1 and 2) and also different from the other maternal half-sibling, progeny 3.

Variation in emissions of volatile chemicals among plant genotypes has been primarily demonstrated in cultivated agricultural plants, such as maize [48], cotton [49], wheat [50], and rice [51], and few studies have reported intraspecific variation in chemical emission in natural populations of plants [52]–[54]. Chemical profile comparisons in this study between half-and full-sibling avocado trees are correlated with the genetic differences. Full-sibling progeny 1 and 2 were grouped together by chemical analysis for all but one location. Chemical profiles from trunks and proximal ends of branches tended to separate from the chemical patterns of the distal ends of branches, petioles and leaves; and all but one location provided good separation among full-and half-siblings. Thus, it should be possible to differentiate siblings using only the chemical emission from the leaves, without damaging the tree by rasping the bark.

In controlled environments or under similar environmental conditions, chemical phenotypes vary in relation with the genetic background of the plant. Assuming that the inbreeding coefficient (F) of the parents of these genotypes is F = 0 (totally non-inbred) then the four maternal half-siblings have a 0.125 probability of having alleles identical by descendant (IBD) at a given locus. That probability increases to 0.25 for the two full-siblings. If F is higher among the parents then the probability of IBD at a given locus is increased. The West Indian race has been found to be much more homozygous than the Guatemalan, and this could be the result of higher amounts of inbreeding [22]. The amount of heterozygosity at the 13 microsatellite loci varied considerably among the parents. For instance “Melendez’, the West Indian × Guatemalan hybrid, was heterozygous at 12 of the 13 loci assayed (92.3%) while the three paternal parents, all of West Indian racial background, were considerably more homozygous with ‘General Bureau’, ‘Wilson Popenoe’, and ‘Waldin’ being 53.8%, 38.4%, and 27.2% heterozygous for these microsatellite loci, respectively. These heterozygosity frequencies were confirmed when compared to a similar study based on 55 microsatellites markers on the same parental genotypes (E.J.B. unpubl.). The four progeny were, on average, heterozygous at 10 of the 13 loci (76.9%). The low heterozygosity of the paternal parents, all West Indian race trees, would suggest that most of the variation in chemical compound expression is derived from the Guatemalan background of the maternal parent ‘Melendez’, a West Indian hybrid × Guatemalan. Therefore, it could be hypothesized from the molecular marker evidence that the variation in chemical phenotype of the progeny studied is due to heterozygosity at loci involved with semiochemical expression.

In the second study, we confirm the possibility to separate the genetic background of two half-sibling avocado populations using their chemical phenotypes. Indeed, in this study, offspring with the same mother were grouped in a cluster according to their paternal heritage based on their volatile chemical profiles. The consistency in the leaf chemical profiles was strong enough to clearly separate trees with closely related genetic backgrounds (full- and half-sibling trees). Chemotaxonomy may be particularly interesting in biogeography and population ecology studies to separate closely related individuals. It might be possible to use chemical analysis of secondary metabolites emitted from the trees to differentiate clones growing in various diverse environments, as their terpenoid emissions are directly influenced by the environmental conditions in which they grow.

In nature, the chemical similarities among closely related siblings could be a problem in terms of host defense against pests. For example, Janzen [55] suggested that tropical specialist pests caused increased mortality of seedlings growing under the canopy of their mother tree. Langenheim and Stubblebine [56] suggested in the same way that if herbivores are adapted to the particular chemical phenotype of an adult tree, they can cause the selective mortality of seedlings with chemical patterns similar to the mother. Thoss and Byers [57] demonstrated that trees killed by bark beetles presented monoterpene composition mostly representative of average monoterpene composition at the considered location, supporting the hypothesis that it is advantageous for an individual to present a different chemical pattern from the norm. The number of potential enemies attacking trees and the related economic losses should be reduced by increasing the genetic diversity, and thus, consequently, increasing the chemical phenotype diversity expressed by the host trees.
